# Erinacine A-Enriched *Hericium erinaceus* Mycelium Delays Progression of Age-Related Cognitive Decline in Senescence Accelerated Mouse Prone 8 (SAMP8) Mice

**DOI:** 10.3390/nu13103659

**Published:** 2021-10-19

**Authors:** Li-Ya Lee, Wayne Chou, Wan-Ping Chen, Ming-Fu Wang, Ying-Ju Chen, Chin-Chu Chen, Kwong-Chung Tung

**Affiliations:** 1 Department of Veterinary Medicine, National Chung Hsing University, Taichung 402204, Taiwan; liya5237@gmail.com; 2 Biotech Research Institute, Grape King Bio Ltd., Taoyuan 325002, Taiwan; Wayne.Chou@grapeking.com.tw (W.C.); WP.Chen@grapeking.com.tw (W.-P.C.); 3 Department of Food and Nutrition, Providence University, Taichung 433303, Taiwan; mfwang@pu.edu.tw; 4 College of Humanities & Social Sciences, Providence University, Taichung 433303, Taiwan; yjchen5@pu.edu.tw; 5 Institute of Food Science and Technology, National Taiwan University, Taipei 106319, Taiwan; gkbioeng@grapeking.com.tw; 6 Department of Food Science, Nutrition and Nutraceutical Biotechnology, Shih Chien University, Taipei 104336, Taiwan; 7 Department of Bioscience Technology, Chung Yuan Christian University, Taoyuan 320314, Taiwan

**Keywords:** *Hericium erinaceus*, senescence accelerated mouse prone 8 (SAMP8), aging, memory

## Abstract

There have been many reports on the neuroprotective effects of *Hericium erinaceus* mycelium, in which the most well-known active compounds found are diterpenoids, such as erinacine A. Previously, erinacine A-enriched *Hericeum erinaceus* mycelium (EAHEM) was shown to decrease amyloid plaque aggregation and improve cognitive disability in Alzheimer’s disease model APP/PS1 mice. However, its effects on brain aging have not yet been touched upon. Here, we used senescence accelerated mouse prone 8 (SAMP8) mice as a model to elucidate the mechanism by which EAHEM delays the aging of the brain. Three-month-old SAMP8 mice were divided into three EAHEM dosage groups, administered at 108, 215 and 431 mg/kg/BW/day, respectively. During the 12th week of EAHEM feeding, learning and memory of the mice were evaluated by single-trial passive avoidance and active avoidance test. After sacrifice, the amyloid plaques, induced nitric oxidase synthase (iNOS) activity, thiobarbituric acid-reactive substances (TBARS) and 8-OHdG levels were analyzed. We found that the lowest dose of 108 mg/kg/BW EAHEM was sufficient to significantly improve learning and memory in the passive and active avoidance tests. In all three EAHEM dose groups, iNOS, TBARS and 8-OHdG levels all decreased significantly and showed a dose-dependent response. The results indicate that EAHEM improved learning and memory and delayed degenerative aging in mice brains.

## 1. Introduction

With the advent of an aging population worldwide, neurodegenerative diseases have become a major socioeconomic concern, of which Alzheimer’s Disease (AD) is most closely tied to aging. Currently, there are no clinically effective drugs that can cure AD. Rather, a reasonable retardation of its progression can be achieved [1]. A preventive angle for treatment is thus preferred, wherein intervention may be done before the diagnosis of mild dementia [2]. A strategy that would be more readily acceptable to the public is dietary supplements that provide preventive effects against the disease. 

The world’s population is aging, with epidemiological studies predicting an increase in the percentage of people over age 60 from 11% presently to 22% in 2050 [3]. Presently, the molecular pathogenesis of AD is not yet completely elucidated, adding to the concern of AD becoming a major health as well as economic crisis [4]. The overarching aims of research on brain aging include, (i) understanding how the brain changes with respect to age, (ii) understanding the causation of these changes, (iii) helping to improve brain health and decrease the detrimental effects of cognitive impairment and neuropsychiatric diseases during the course of aging [5,6,7].

SAMP8 is a proven animal model used to study the genetic mechanisms behind learning and memory impairment in the life cycle. For example, it has been used to show that excess amyloid aggregation in the early stages of life causes learning and memory impairment and disability [6,7]. The lion’s mane mushroom (*Hericeum erinaceus*) is a popular species of edible mushroom in Eastern countries, such as China, Japan, Malaysia, Singapore and Taiwan, where they are consumed for both their culinary and medicinal values. Research on its putative benefits is mainly divided into two directions. Firstly, their polysaccharides have been revealed to possess tumor suppressive and immune boosting qualities [8,9,10]. The second direction of interest is diterpenoids, including the erinacines found only in liquid cultured mycelium, which have been found to stimulate glial cells to excrete nerve growth factor (NGF) [11,12,13,14]. In 2005, Japanese researchers conducted the first animal trials using erinacine A, showing its ability to increase NGF levels in the locus coeruleus and the hippocampus. This confirms erinacine A affects the central nervous system [15].

In recent years, there has been a considerable amount of research from Taiwan into the effects of Erinacine A-enriched *Hericium erinaceus* mycelia (EAHEM) in protecting against neurological disorders. In an animal model of ischemic stroke, feeding with 50 and 300 mg/kg body weight of EAHEM reduced the ratio of cerebral infarction by approximately 22 and 44 percent, respectively [16]. In an MPTP-induced model of Parkinson’s disease, feeding low doses of EAHEM increased dopamine and NGF levels in the brain approximately 1.82- and 1.58-fold, respectively [17]. Five-month-old APP/PS1 transgenic mice fed with EAHEM at a dose of 300 mg/kg/day for 30 days were found to possess decreased Aβ plaque burden, increased NGF/proNGF ratio and increased insulin degrading enzyme (IDE) levels. In the nest building assay, feeding of EAHEM yielded significant differences in score and unshredded cotton [18].

The above animal studies confirmed the protective effects of EAHEM on the brain in both neurotoxin-induced and physically-induced brain disorders. However, direct beneficial effects in the setting of brain degeneration as a result of aging has not been studied before. Therefore, this study aims to assess the brain aging, learning and memory conditions in an aging mouse model fed with EAHEM.

## 2. Materials and Methods

### 2.1. Experimental Animals

The animals used in this study were 3-month-old male and female senescence accelerated mice (SAMP8). Mice were housed in clear plastic cages of 30 cm (W) by 20 cm (D) by 10 cm (H) size. Mice were kept in a clean room maintained at a controlled temperature of 25 ± 2 °C, relative humidity of 65 ± 5% and with automatically controlled light-dark cycle. The dark period lasted from 07:00 to 19:00, and the light period was from 19:00 to 07:00. Feed and water were given ad libitum. SAMP8 mice used in this study were an age-accelerated strain of mice developed by the University of Kyoto. The animals in this study were bred and kept at Ching-Yi University. Experiments in this study were approved by the IACUC (#20120918-P04). Mice were randomly housed in groups of 4 per cage.

### 2.2. Mycelium Preparation

*H*. *erinaceus* strain (BCRC 35669) was obtained from the Bioresources Collection and Research Center (BCRC) in Food Industry Research and Development Institute (Hsinchu, Taiwan). The seed cultures were grown in 2 L flasks containing 1.3 L of synthetic medium (4.5% glucose, 0.5% soybean powder, 0.25% yeast extract, 0.25% peptone and 0.05% MgSO_4_, adjusted to pH 4.5) Scale-up from a shake flask to 500 L fermenters and 20 ton fermenters lasted for 5 days and 12 days, respectively. At the end of the fermentation process, the mycelia were then harvested, lyophilized, grounded to a powder and stored in a desiccator at room temperature. Cultivation and liquid fermentation of Lion’s mane mushroom (*H. erinaceus*) mycelium used in this study were conducted, as described previously. The concentration of Erinacine A in mycelium was determined to be 5 mg/g, using the HPLC method described previously [19].

### 2.3. Experimental Design

In the test, three-month-old SAMP8 mice were grouped, with 20 females and 20 males, as follows: control, low dose group (108 mg/kg/bw/day), intermediate dose group (215 mg/kg/bw/day), high dose group (431 mg/kg/bw/day) EAHEM daily for 13 weeks.

### 2.4. Behavioral Tests

#### 2.4.1. Passive Avoidance Task

Single-trial passive avoidance test was conducted in a 35 (W) by 17 (D) by 20 (H) aluminum shuttle cage (Coulbourn instruments Model E10-15), divided into illuminated and dark compartments and mutually accessible via a 7.5 (W) by 6.5 (D) guillotine door (Coulbourn instruments Model E10-15GD). The bottom of the chamber was lined with parallel metal rods 1 cm apart, coupled to an electric emitter to administer foot shock. At the start of the test, mice were placed in the illuminated chamber, after allowing 10 s of environment familiarization, the door to the dark chamber was opened to allow the mouse to freely explore. The dark chamber was naturally favorable to the nocturnal mice. Once the mice entered the dark chamber, the door was quickly closed and a 0.5 μA 0.5 s foot shock was delivered three consecutive times, each 5 s apart, thus completing the training session. The memory of the mice was assessed 1 day, 2 days and 3 days after this training step by conducting the same procedure, albeit without delivering any foot shock. The latency time was recorded with total assessment time not exceeding 180 s. Increased latency time indicates better memory in the mice.

#### 2.4.2. Active Shuttle Avoidance Test

The same shuttle cage was used to set up an active shuttle avoidance test. In this test, mice were placed in one chamber for an inter-trial interval of 10 s. Subsequently, a light and sound conditioned stimulus (CS) was presented for 10 s. If during CS presentation, the mice still stayed in the same chamber, a 5 s 0.3 mA foot shock was delivered automatically, which serves as the unconditioned stimulus (UCS). If the mice entered the adjacent chamber under CS presentation, foot shock was not delivered. The software presented CS or UCS according to the mice’ reaction. Each mouse underwent one session consisting of 5 turns of CS/UCS assessment before being put back into its cage. After 15–20 min of down time, the above CS/UCS session was repeated 4 times each day. This was done for 4 consecutive days. Ultimately, the number of avoidance responses to CS were counted, with fewer avoidance responses indicating relatively weaker memory. The above tests were used to assess the effect of different doses of EAHEM on the learning and memory of mice.

### 2.5. Tissue Preparation

After mice were sacrificed, the brain was removed and fixed in 10% neutral formaldehyde solution for approximately one week. The method for slicing midbrain tissue was adapted from Shimada et al.’s method [20]. The brain was divided into 6 sectors. The β-amyloid aggregation area ratio was measured. Computer image processing system Leica Q500 (Ernst Leitz Wetzlar, Wetzlar, Germany) was used to calculate under 20× magnification. The formula was described as: (β-amyloid aggregation area/whole brain area) × 100%.

### 2.6. Measurement of iNOS and TBARS

Mice were sacrificed by decapitation, and the brain tissue was extracted according to the method by Glowinski and divided into the cortex and hippocampus [21]. The hippocampus was homogenized after the addition of 1 mL phosphate buffered saline (PBS), pH 7.4 buffer, then centrifuged at 15,000× *g* for 30 min at 4 °C. The supernatant was kept as a liquid and preserved at −80 °C until needed, then it was thawed and centrifuged (12,000× *g*, 5 min, 4 °C). This was collected for the measurement of iNOS by using ELISA kits purchased from USCN (USCN Business Co., Ltd., Wuhan, China). For analysis of the levels of thiobarbituric acid reactive substances (TBARS) in mouse brain, 100 μL of homogenized brain supernatant was mixed with 100 μL sodium dodecyl sulfate (SDS) solution and 4 mr regent for an hour. The mixture was centrifuged at 1600× *g* under 4 °C for 10 min, then read the value at 535 nm.

### 2.7. Data Analysis

The data in this study were analyzed by SigmaPlot 10.0 data analysis package. The data are all expressed as mean ± SEM. The data were analyzed by one-way analysis of variance (ANOVA), to determine the difference between groups. Duncan’s multiple range test was used to compare differences between groups. *p* < 0.05 is considered significantly different.

## 3. Results

### 3.1. Behavioral Assays

**Figure 1:**
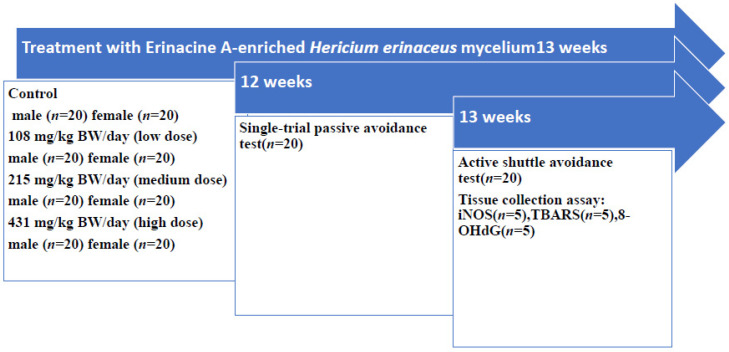
Schematic representation of the experimental design.

The experiment design for this study is presented in Figure 1. Briefly, male and female SAMP8 mice were fed different doses daily. After 12 weeks of feeding, the latency time in the single-trial passive avoidance test was recorded. The results indicated that in both male and female SAMP8 mice, there was no significant difference between control and treated groups in acquisition training. However, 1 and 2 days after training, latency time was recorded to be significantly higher (*p* < 0.05) in treated groups. During this time, daily EAHEM feeding was not ceased. Latency time displayed a decreasing trend in all groups 3 days after training. This may be due to the absence of foot shock in the tests following the first day of the experiment. The latency time lasted longer 1 and 2 days after training, but due to the decrease in memory retention with elapsed time, latency time consequently decreased. The single-trial passive avoidance test results indicated that feeding EAHEM enhanced the learning and memory of mice (Table 1).

Active shuttle avoidance test of SAMP8 mice was conducted after 13 weeks of EAHEM feeding. The average number of avoidance responses recorded during the test was positively correlated to learning. On the first day of the trial, as the mice were still in the training phase, there was no significant difference between avoidance responses of each group. On the 2nd, 3rd and 4th day of the trial, EAHEM-fed groups of male and female mice recorded a significantly higher number of avoidance responses (*p* < 0.05) compared to the control group (Table 2). This indicates that while 12 weeks of feeding with EAHEM did not improve the performance of the mice on the day of training, they were able to retain this training memory afterwards. The results from the two sets of learning memory trials indicate that EAHEM-fed test groups displayed significantly improved learning and memory.

### 3.2. Comparison of Brain Pathological Markers

**Figure 2:**
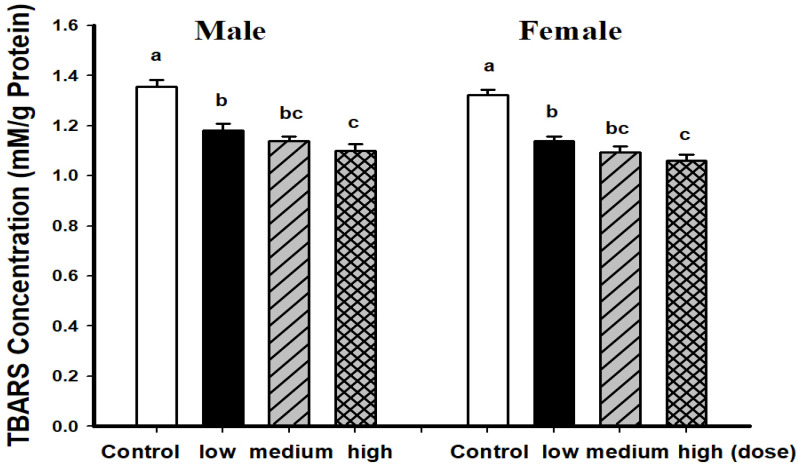
Thiobarbituric acid reactive substances (TBARS) levels in mice brain after 13 weeks feeding. Low dose group (108 mg/kg/bw/day), intermediate dose group (215 mg/kg/bw/day), high dose group (431 mg/kg/bw/day). Values are expressed as mean ± S.E.M. and analyzed by one-way ANOVA (*n* = 10). Groups with different letters denote significant difference between each group (*p* < 0.05).

**Figure 3:**
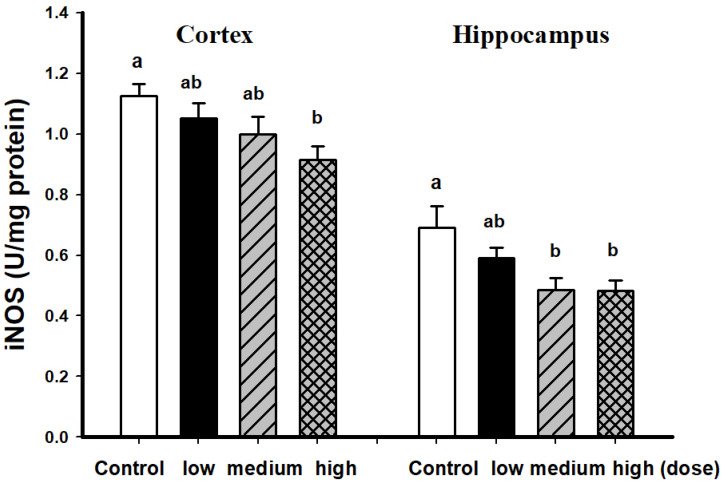
iNOS activity in male mice brain after 13 weeks feeding. Low dose group (108 mg/kg/bw/day), intermediate dose group (215 mg/kg/bw/day), high dose group (431 mg/kg/bw/day). Values are expressed as mean ± S.E.M. and analyzed by one-way ANOVA (*n* = 5). Groups with different letters denote significant difference between each group (*p* < 0.05).

Male and female mice fed with EAHEM possessed significantly lowered brain TBARS levels (*p* < 0.05), with a high dose being the most effective. This shows that after 13 weeks of EAHEM feeding, the brain TBARS levels can be lowered (Figure 2). Male mice fed with a high dose of EAHEM possessed approximately 1.21-fold lower cortical iNOS when compared with control group (*p* < 0.05). At the same time, iNOS activity in the hippocampus was approximately 1.43-fold lower in mice fed with a medium and high dose of EAHEM (Figure 3) compared with the control group.

**Figure 4:**
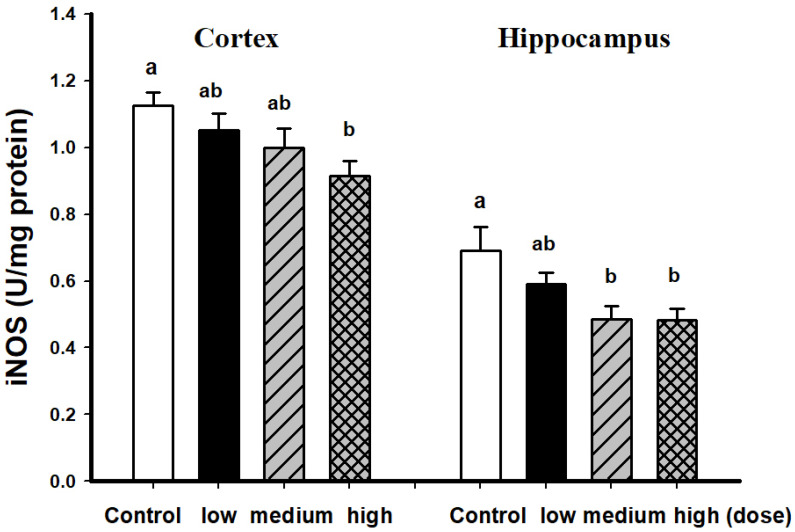
iNOS activity in female mice brain fed after 13 weeks feeding. Low dose group (108 mg/kg/bw/day), intermediate dose group (215 mg/kg/bw/day), high dose group (431 mg/kg/bw/day). Values are expressed as mean ± S.E.M. and analyzed by one-way ANOVA (*n* = 5). Groups with different letters denote significant difference between each group (*p* < 0.05).

In female SAMP8 mice fed with EAHEM for 13 weeks, the cortical iNOS levels were found to be significantly lower than the control group, up to 1.93-fold decrease when comparing the high dose and control group, while the hippocampus iNOS levels were 1.44-fold lower (Figure 4).

**Figure 5:**
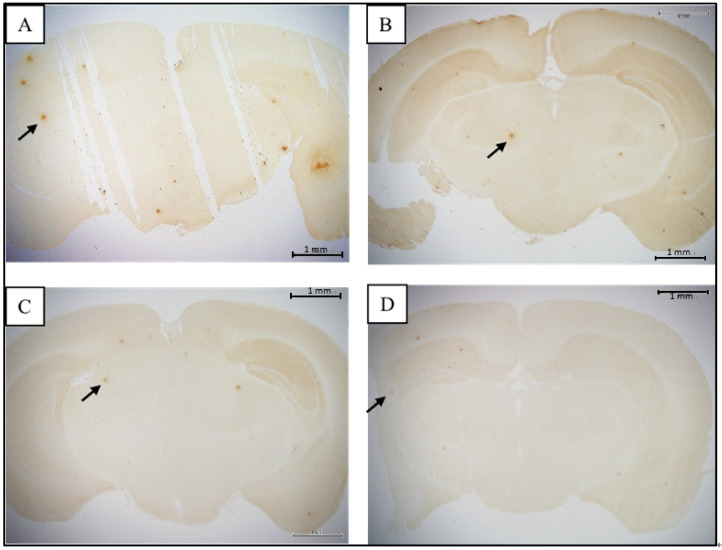
β-amyloid plaque in male mice (20× magnification). (**A**) = control (ddH_2_O), (**B**) = low dose (108 mg/kg BW/day), (**C**) = medium dose (215 mg/kg BW/day), (**D**) = high dose (431 mg/kg BW/day) (*n* = 5). Arrows indicate β-amyloid plaque.

**Figure 6:**
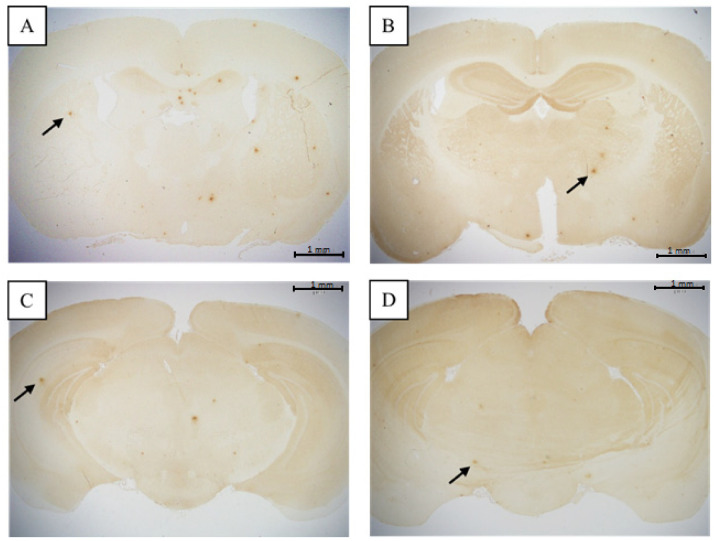
β-amyloid plaque in female mice (20× magnification). (**A**) = control (ddH_2_O), (**B**) = low dose (108 mg/kg BW/day), (**C**) = medium dose (215 mg/kg BW/day), (**D**) = high dose (431 mg/kg BW/day) (*n* = 5). Arrows indicate β-amyloid plaque.

The histological section of control and EAHEM-fed group were observed under light microscope at 20× magnification to evaluate alpha amyloid aggregation (Figure 5 and Figure 6). The tissue slice size was 20 × 50 mm in length and width, and the appropriate thickness was 5 mm.

**Figure 7:**
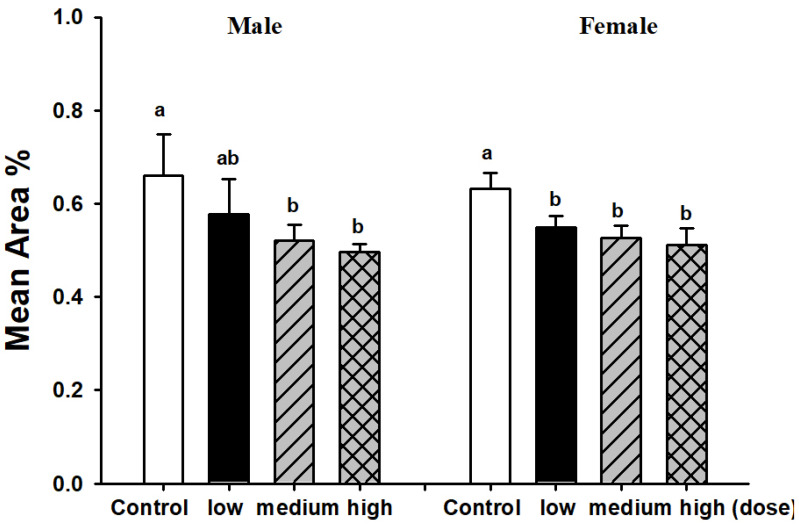
Mean area of β-amyloid plaque in male mice brain after 13 weeks feeding. Low dose group (108 mg/kg/bw/day), intermediate dose group (215 mg/kg/bw/day), high dose group (431 mg/kg/bw/day). Values are expressed as mean ± S.E.M. and analyzed by one-way ANOVA (*n* = 5). Groups with different letters denote significant difference between each group (*p* < 0.05).

**Figure 8:**
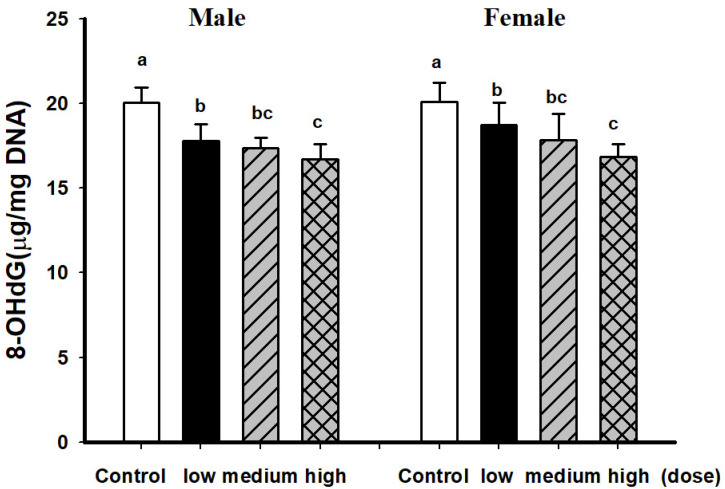
8-OHdG levels of mice brain after 13 weeks feeding. Low dose group (108 mg/kg/bw/day), intermediate dose group (215 mg/kg/bw/day), high dose group (431 mg/kg/bw/day). Values are expressed as mean ± S.E.M. and analyzed by one-way ANOVA (*n* = 10). Groups with different letters denote significant difference between each group (*p* < 0.05).

Medium and high dose EAHEM-fed mice showed a significantly lower percentage in the brain Aβ plaque number (*p* < 0.05), by 21.1% and 24%, respectively. Low, medium and high dose EAHEM-fed mice showed a lower percentage of Aβ aggregation area (*p* < 0.05) by 12.7%, 15.9% and 19.0%, respectively (Figure 7). 8-hydroxy-2’-deoxyguanosine (8-OHdG) is an indicator of mitochondrial DNA damage [22]. Therefore, we chose to measure 8-OHdG levels in mice brain and found them to be significantly lower in EAHEM-fed groups (*p* < 0.05). The high dose group showed the greatest negative difference in 8-OHdG levels at 83.5% and 83.7%, compared to that of control, in male and female mice, respectively (Figure 8).

## 4. Discussion

Aging is known to be associated with cognitive impediment. The biochemical and physiological basis is still not clear, but the hippocampus is known to play an important role in cognitive function. Kumar et al. utilized an array technique to probe how hippocampus genes affect cognitive impairment in SAMP8 mice of different ages, finding significant differences in anti-oxidative and xenobiotic metabolism gene expression [23]. SAMP8 mice displayed learning and memory deficits as young as 4 months of age, as well as further time-dependent decline [24]. The results of the active avoidance and passive avoidance test in this study demonstrate that long-term intake of EAHEM can improve learning as well as memory retention. Previously, a similar recovery of cognitive decline in APP/PS1 transgenic mice conferred by erinacine A was demonstrated by Tzeng et al. using a Morris water maze, wherein mice fed with erinacine A displayed shorter escape latency [25].

Brain neurons and endothelial cells produce nitric oxide (NO) as a product of L-arginine and oxygen by nitric oxide synthase, which act as signaling molecules in the brain. There are three isoforms of nitric oxide synthase, endothelial NO synthase (eNOS), neuronal NO synthase (nNOS) and inducible NO enzyme (iNOS). In most cells such as glial cells, macrophages, skeletal muscle, neurons, platelets and white blood cells, iNOS expression is elevated in response to inflammation and oxidative pressure [26]. In this study, feeding EAHEM to mice lowered iNOS expression in the brain, implying that its protective effect is conferred by directly lowering oxidative stress or inflammation. This is in agreement with Lee et al.’s study, wherein the feeding of *H. erinaceus* mycelium or erinacine A correlated with the attenuation of p38/MAPK and C/EBP homologous protein (CHOP), downregulation of iNOS and nitrotyrosine-containing proteins, resulting in reduction of inflammation, increased free radical scavenging and protection from ischemic brain damage [16].

More and more evidence points toward the long-term accumulation of peroxidation of DNA, proteins and lipids by free radicals as the culprit of the aging brain and functional degeneration [27]. Four age-related diseases have been pathologically linked to free radical reactions, including cancer, atherosclerosis, hypertension and amyloidosis. Regular consumption of foods that inhibit free radical reactions, such as dietary fibers can decrease endogenous free radical reactions, consequently lengthening the patient’s life span by five or more years as well as leading to a healthier life [28]. In our study, TBARS levels in the brain were indeed measured to be decreased, indicating decreased lipid peroxidation. Previous studies on 13 AD patients compared to 10 control subjects found that TBARS levels were higher for AD patients in all parts of the brain, except the middle frontal gyrus [29]. Free radicals have also been shown to cause damage to brain lipids, carbohydrates, proteins and DNA, which is highly correlated to neuronal death in neurodegenerative diseases. Peroxidation of lipids in the brain causes poly-unsaturated fatty acid (PUFA) levels to drop, and 4-hydroxynonenal (4-HNE), a neurotoxic aldehyde product of PUFA oxidation, to rise [30].

The anti-oxidative stress capacity of EAHEM has also been reported previously in an MPTP-induced model of the increase in oxidative stress and consequent dopaminergic neuronal cell death. Feeding EAHEM was found to lower nitrotyrosine and 4-HNE expression while also recovering motor ability in a mouse rotarod test. The mechanism is that erinacine A treatment provides protection against the endoplasmic reticulum stress caused by neurotoxicity and neuronal cell apoptosis mainly via activating RE1α/TRAF2, JNK1/2 and p38 MAPK pathways, thus allowing expression of CHOP, IKB-β and NF-κB, as well as Fas and Bax [31]. Shimbo et al. observed that the feeding of erinacine A at 8 mg/kg/day to rats increased catecholamine and NGF [15].

AD is a chronic neurodegenerative disease that primarily affects the medial temporal lobe and the neocortical structures. The pathological markers of AD include neuronal plaques and neuronal entanglement, the former of which is caused by amyloid-β peptide aggregation and the latter caused by hyperphosphorylation of microtubular tau protein in neurons that leads to changes in the cellular structure [32]. Aβ, as oligomers, are neurotoxic, causing damage to cellular signal transduction, including impaired synaptic plasticity, increased tau protein phosphorylation, increased GSK-3β activity, imbalance of calcium homeostasis causing neuronal cell death and apoptosis. Aβ also interacts with neurons and neuroglial cells, activating inflammation, mitochondrial dysfunction and increasing oxidative pressure [33,34]. Currently, two types of proteins have been identified as being closely linked to the removal of Aβ in the brain: apolipoprotein E (ApoE) and insulin-degrading enzyme (IDE). Although the mechanism of action is not currently confirmed, ApoE has been reported to interact with Aβ to inhibit its polymerization or chaperone its proteolysis, while IDE has been shown to degrade soluble Aβ extracellularly [35,36,37]. Previous studies indicated that feeding EAHEM to APP/PS1 mice showed increased IDE expression and decreased glial and microglial cell activation, both of which are possible factors that may lower Aβ plaque burden [18]. A similar downward trend in Aβ plaque percentage is present in our study. Previously, Chien-Chih et al. also proved that erinacine A increased IDE levels to twice that of the control group in APP/PS1 mice [25].

Reactive oxygen species (ROS) are products of many processes, such as arachidonic acid metabolism, lipid peroxidation and phagocyte activation [38]. During ischemia-reperfusion in the brain, 8-OHdG is generated from a DNA base modified by ROS [39]. Luceri et al. evaluated serum ROS, 8-OHdG and DNA repair enzyme levels in very young (2 months old), young (8 months old) and middle aged (15 months old) mice, revealing that a decrease in DNA repair capabilities coincided with elevated 8-OHdG in older mice [40]. 8-OHdG is also related to neurodegenerative diseases, as it is a good diagnostic marker found in the cerebrospinal fluid of Parkinson’s disease patients [41]. In a study on major depressive disorder (MDD) and patients’ inflammatory and oxidative stress-related responses to an antidepressant drug, those who responded poorly to the drug possessed higher levels of F2-isoprostanes prior to treatment, while 8-OHdG and IL-6 were both associated with antidepressant response [42]. There is some evidence that 8-OHdG immunoreactivity may not be an ideal evaluation method of infarction injury. In Nogami et al.’s study, age was found to be negatively correlated to 8-OHdG immunoreactivity in glial cells of autopsied humans. There was no significant difference in 8-OHdG reactivity between glial cells in the area immediately surrounding the infarction and the non-ischemic areas [43]. In Chiu et al.’s study, ethanolic extract of EAHEM was given to mice before challenged with repeated restraint stress (RS), tail suspension test and forced swimming test to evaluate its impact on depression behavior and serum markers. Serotonin and dopamine levels were found to be significantly elevated, while inflammatory markers IL-6 and TNF-α were both significantly lowered. The mechanism was demonstrated to be the activation of the BDNF/TrkB/PI3K/Akt/GSK-3 pathways to inhibit NF-κB pathway [44]. The literature above strongly supports that EAHEM decreases inflammation and at the same time, prevents oxidative stress to confer anti-aging functions.

## 5. Conclusions

This study demonstrates that EAHEM can lower oxidative stress in the brain and further prevent chronic inflammation, consequently decreasing amyloid aggregation and improving learning and memory. Future efforts will focus on elucidating the mechanism, and possibly achieving the inhibition of amyloid generation, enhancement of amyloid clearance or neuronal regeneration.

## Figures and Tables

**Table 1:** Single-trial passive avoidance latency time in mice after 12 weeks feeding.

	Trial	1 Day	2 Days	3 Days
Male				
Control	48.20 ± 0.95	58.55 ± 1.18 ^a^	56.35 ± 1.22 ^a^	49.25 ± 0.86
108 mg/kgBW/day	49.65 ± 0.94	67.98 ± 0.80 ^b^	61.00 ± 0.79 ^b^	51.50 ± 0.82
215 mg/kgBW/day	47.20 ± 0.86	69.70 ± 1.11 ^b^	62.20 ± 0.83 ^b^	51.25 ± 1.34
431 mg/kgBW/day	47.40 ± 0.91	70.80 ± 0.87 ^b^	63.35 ± 0.85 ^b^	52.60 ± 1.56
Female				
Control	47.60 ± 1.18	60.65 ± 0.86 ^a^	55.80 ± 1.18 ^a^	49.10 ± 1.01
108 mg/kgBW/day	48.55 ± 1.42	70.00 ± 0.78 ^b^	64.40 ± 1.15 ^b^	51.85 ± 1.11
215 mg/kgBW/day	44.35 ± 1.46	71.90 ± 0.82 ^b^	65.85 ± 0.67 ^b^	52.65 ± 2.07
431 mg/kgBW/day	46.30 ± 1.57	72.20 ± 0.79 ^b^	64.85 ± 0.67 ^b^	48.45 ± 1.86

Values are expressed as mean ± S.E.M. and analyzed by one-way ANOVA (*n* = 20). Groups with different letters denote significant difference between each group (*p* < 0.05).

**Table 2:** Active shuttle avoidance test in mice after 12 weeks feeding.

	Day 1	Day 2	Day 3	Day 4
Male				
Control	9.85 ± 0.34	12.25 ± 0.36 ^a^	11.55 ± 0.36 ^a^	12.75 ± 0.31 ^a^
108 mg/kgBW/day	10.35 ± 0.44	14.50 ± 0.51 ^b^	13.85 ± 0.51 ^b^	14.30 ± 0.56 ^b^
215 mg/kgBW/day	10.00 ± 0.43	14.90 ± 0.45 ^b^	14.70 ± 0.45 ^b^	15.05 ± 0.39 ^b^
431 mg/kgBW/day	9.95 ± 0.32	14.05 ± 0.40 ^b^	14.70 ± 0.40 ^b^	15.00 ± 0.24 ^b^
Female				
Control	12.35 ± 0.57	11.05 ± 0.40 ^a^	12.30 ± 0.25 ^a^	13.70 ± 0.19 ^a^
108 mg/kgBW/day	10.70 ± 0.44	12.85 ± 0.37 ^b^	14.55 ± 0.37 ^b^	15.35 ± 0.46 ^b^
215 mg/kgBW/day	10.45 ± 0.32	13.85 ± 0.31 ^b^	15.55 ± 0.46 ^b^	15.40 ± 0.20 ^b^
431 mg/kgBW/day	10.20 ± 0.19	13.50 ± 0.53 ^b^	15.10 ± 0.37 ^b^	15.35 ± 0.34 ^b^

Values are expressed as mean ± S.E.M. and analyzed by one-way ANOVA (*n* = 20). Groups with different letters denote significant difference between each group (*p* < 0.05).

## Data Availability

All data can be assessed from L.Y. Lee via the email address.
